# Immunoadjuvant and Humoral Immune Responses of Garlic (*Allium sativum* L.) Lectins upon Systemic and Mucosal Administration in BALB/c Mice

**DOI:** 10.3390/molecules27041375

**Published:** 2022-02-17

**Authors:** Shruthishree D. Padiyappa, Hemavathi Avalappa, Madhusudana Somegowda, Shankarappa Sridhara, Yeldur P. Venkatesh, Bettadatunga T. Prabhakar, Siddanakoppalu N. Pramod, Mona S. Almujaydil, Shadi Shokralla, Ashraf M. M. Abdelbacki, Hosam O. Elansary, Ahmed M. El-Sabrout, Eman A. Mahmoud

**Affiliations:** 1 Food Allergy and Immunology Laboratory, Department of Studies in Food Technology, Davangere University, Shivagangotri, Davangere 577 007, India; shruthigopal.sg.21@gmail.com (S.D.P.); hemavathia02@gmail.com (H.A.); 2 Molecular Biomedicine Laboratory, Postgraduate Department of Biotechnology, Sahyadri Science College, Kuvempu University, Shivamogga 577 203, India; pbtssc@gmail.com; 3 Department of Plant Biochemistry, University of Agriculture and Horticulture Science, Shivamogga 577 204, India; ysmadhu84@gmail.com; 4 Center for Climate Resilient Agriculture, University of Agriculture and Horticulture Science, Shivamogga 577 204, India; sridharas1968@gmail.com; 5 Department of Biochemistry and Nutrition, CSIR–Central Food Technological Research Institute (CFTRI), Mysuru 570 020, India; venkatyp@yahoo.com; 6 Department of Food Science and Human Nutrition, College of Agriculture and Veterinary Medicine, Qassim University, Buraydah 51452, Saudi Arabia; m.almujaydil@qu.edu.sa; 7 Centre for Biodiversity Genomics, University of Guelph, Guelph, ON N1G 2W1, Canada; sshokral@uoguelph.ca; 8 Applied Studies and Community Service College, King Saud University, Riyadh 11451, Saudi Arabia; abdelbacki1978@yahoo.com; 9 Plant Production Department, College of Food & Agriculture Sciences, King Saud University, Riyadh 11451, Saudi Arabia; 10 Department of Applied Entomology and Zoology, Faculty of Agriculture (EL-Shatby), Alexandria University, Alexandria 21545, Egypt; elsabroutahmed@alexu.edu.eg; 11 Department of Food Industries, Faculty of Agriculture, Damietta University, Damietta 34511, Egypt; emanmail2005@yahoo.com

**Keywords:** adjuvanticity, agglutinin, garlic lectins, immunogenicity, immunomodulatory, BALB/c mice, ASA I and II, ovalbumin

## Abstract

Dietary food components have the ability to affect immune function; following absorption, specifically orally ingested dietary food containing lectins can systemically modulate the immune cells and affect the response to self- and co-administered food antigens. The mannose-binding lectins from garlic (*Allium sativum* agglutinins; ASAs) were identified as immunodulatory proteins in vitro. The objective of the present study was to assess the immunogenicity and adjuvanticity of garlic agglutinins and to evaluate whether they have adjuvant properties in vivo for a weak antigen ovalbumin (OVA). Garlic lectins (ASA I and ASA II) were administered by intranasal (50 days duration) and intradermal (14 days duration) routes, and the anti-lectin and anti-OVA immune (IgG) responses in the control and test groups of the BALB/c mice were assessed for humoral immunogenicity. Lectins, co-administered with OVA, were examined for lectin-induced anti-OVA IgG response to assess their adjuvant properties. The splenic and thymic indices were evaluated as a measure of immunomodulatory functions. Intradermal administration of ASA I and ASA II had showed a four-fold and two-fold increase in anti-lectin IgG response, respectively, vs. the control on day 14. In the intranasal route, the increases were 3-fold and 2.4-fold for ASA I and ASA II, respectively, on day 50. No decrease in the body weights of animals was noticed; the increases in the spleen and thymus weights, as well as their indices, were significant in the lectin groups. In the adjuvanticity study by intranasal administration, ASA I co-administered with ovalbumin (OVA) induced a remarkable increase in anti-OVA IgG response (~six-fold; *p* < 0.001) compared to the control, and ASA II induced a four-fold increase vs. the control on day 50. The results indicated that ASA was a potent immunogen which induced mucosal immunogenicity to the antigens that were administered intranasally in BALB/c mice. The observations made of the in vivo study indicate that ASA I has the potential use as an oral and mucosal adjuvant to deliver candidate weak antigens. Further clinical studies in humans are required to confirm its applicability.

## 1. Introduction

Lectins are typical globular proteins of nonimmune origin displaying binding to specific carbohydrates of oligo/polysaccharides, glycoproteins, and glycolipids. Among the many biological effects of dietary lectins, their immunomodulatory effects have been well studied [1,2,3]. Plant lectins are generally resistant to breakdown by the digestive enzymes and have been shown to alter the intestinal epithelium; following absorption, lectins get internalized, and some appear in their native form in the blood [4,5]. Lectins promote diverse biological consequences and enhance the absorption of dietary co-administered antigens [6,7], and on oral administration, these lectins are capable of inducing specific targeted immune responses to self- and co-administered antigens, which is in contrast to nonfunctional dietary food proteins [8].

Mucosal or oral administration of food antigens is considered not effective in provoking robust and long-lasting immune responses; these require high doses, and the response induced is potentially for short duration [9]. The adjuvant-tagged antigen delivery systems can target the antigen to the mucosal epithelium and enhance immune recognition by protecting the antigen, which elicits a specific immune response to the administered antigen [10]. The dietary food lectins, which are capable of specifically recognizing and binding to mucosal gut epithelial cells, are identified to be stable and intact after feeding and ingestion in the form of food [8,11]; they are also shown to selectively bind to mucosa-associated epithelial cells (M cells) in the mouse gut-associated lymphoid tissue (GALT) and Peyer’s patches [12]. Evidence has been exhibited for the dislocation of dietary food lectins across the gut epithelium in both humans and mice [8,13]. This can be exploited for the formulation of a lectin-based oral vaccine-targeted delivery to induce both mucosal and systemic immune responses against weak antigens. Several plant food lectins are known to be mitogens, to activate lymphocytes in vitro [14,15], and to induce high levels of specific anti-lectin serum IgG through the mucosal and oral routes of administration in mice [16,17].

The dietary proteins with lectin or lectin-like properties are well established to interact with immune cells and are able to induce a strong IgG mediated humoral immune response through the generation of anti-lectin antibodies when delivered through mucosal or oral routes [18,19,20,21]. However, the oral or mucosal delivery of certain nonreplicating weak antigens does not have the capability to trigger a strong immune response. This requires repetitive multiple doses through the mucosal route, and sometimes the response may result in a systemic unresponsiveness [22]. A range of molecules, like bacterial vectors, liposomes, and biodegradable microparticles, are employed to mucosally delivered vaccines to enhance the immune responses [21,22]. To date, the potent mucosal adjutants, which were recognized for the delivery of co-administered weak antigens through the mucosal route, are heat-labile enterotoxin (LT), mistletoe lectin (ML-1), and cholera toxin (CT) [19,23,24]. Although the toxicity and systemic physiological effects of the adjuvant components prevent clinical applications, the molecules with retained mucosal adjuvanticity and with low toxicity have generated interest to induce an increased immune response to co-administered antigens [25,26,27].

Garlic (*Allium sativum* L., Amaryllidaceae family) [28] is present in many prepared/processed foods and food products as a flavor enhancer, and none of its components are included in any vaccine preparations or medicines administered to humans. The immunomodulatory activities of garlic have been well established [29]. Notably, garlic lectins or agglutinins (ASA I, ASA II) purified from garlic bulbs with hemagglutinating and leucoagglutinating properties have been confirmed as the principal immunomodulatory proteins in garlic [14,15,30]. Garlic lectins show weak binding to mannose but display strong binding to oligomannosides and also high-mannose *N*-glycans [31]. *Allium sativum* agglutinins (ASA I and II) have been isolated and purified from raw garlic bulbs and their in vitro immunomodulatory activities on lymphocytes and phagocytes have been studied earlier [11,15]. There is no available report on the mucosal immunogenicity or adjuvanticity properties of garlic lectins. The garlic lectins are not known for the ability to induce an immune response against self- or co-administered antigens. The present study reports the investigation on the effect of garlic lectins (ASA I and II) on the humoral anti-lectin antibody response and the humoral adjuvant response for a known weak antigen, ovalbumin (OVA), either by the systemic (intradermal) or mucosal (intranasal) routes of co-administration and immunization in BALB/c mice.

## 2. Materials and Methods

### 2.1. Garlic Sample, Reagents, and Chemicals

Concanavalin A (Con A), bovine serum albumin (BSA), ovalbumin (OVA; type V, hen egg), and alkaline phosphatase (ALP)-conjugated goat anti-mouse IgG were obtained from Sigma-Aldrich, St. Louis, MO, USA. The 96-welled, flat-bottomed, and conical-shaped microtiter plates (MICROLON) were the product of Greiner Bio-One GmbH, Frickenhausen, Germany. All other experimental reagents and chemicals used in this study were of analytical grade and were procured from HiMedia and SRL, India. The garlic (*Allium sativum* L.) plant is listed in ‘The Plant List’ website: http://www.theplantlist.org/tpl1.1/record/kew-296499 (accessed on 19 October 2018). A specimen of the dried garlic bulb and the garlic plant were deposited in the plant herbarium (Department of Studies in Botany and Seed Technology, Sahyadri Science College, Constituent College of Kuvempu University, Shivamogga, Karnataka State, India) for authentication of the garlic bulb (voucher no. KU/SSC/BOT/209/1/2017-18) and the garlic plant (voucher no. KU/SSC/BOT/209/2/2017-18) dated 10 November 2017.

### 2.2. Experimental Animals

The following study was undertaken after obtaining the clearance of the Institutional Animal Ethics Committee (IAEC approval #235/12). Six- to seven-week-old female BALB/c mice weighing around 22–25 g was obtained from the Central Animal Facility of the Indian Institute of Science (IISc), Bengaluru, India; they were maintained in the special room provided in the animal house facility present at the Department of Biochemistry and Nutrition, CFTRI, Mysuru. The experiments and research plan related to the use and care of the BALB/c mice complied with all the relevant institutional policies and national regulations. Mice had access to a special commercial diet (pellet) comprising primarily of cereal products and were housed as a group of six in cages. The ambient room conditions were maintained with a temperature at 23 ± 3 °C with a relative humidity of 50 ± 5% and a 10–12 h period of light/dark cycle.

### 2.3. Preparation of Raw Garlic Extracts (RGE) and Purification of Garlic Lectins

Raw garlic extract (RGE) was prepared from garlic bulbs. A total of 25 g of fresh garlic was obtained from the market and peeled to remove the bulb coats. Then, they were washed, smashed, and suspended in 50 mL of phosphate-buffered saline (PBS, pH 7.4). Further, it was homogenized by blending to obtain a 50% *w*/*v* raw garlic extract. The extract was placed at 4 °C for 2 h to increase the protein extraction process with continuous stirring. Later, it was initially passed through porous gauze and then filtered through a muslin cloth to obtain the clear filtrate. The filtrate was further centrifuged at 10,000 rpm for 20 min at 4 °C. The pale yellowish clear supernatant obtained after centrifugation was designated as RGE and stored in aliquots at 4 °C.

Garlic agglutinins (ASA I and II) were purified from the raw garlic extract (RGE) obtained from the bulbs, essentially as described earlier [15]. Briefly, a 1:10 *w*/*v* extract of freshly peeled garlic bulbs was made in 20 mM 1,3-diaminopropane, and the resulting extract was processed by ultrafiltration (Amicon Stirred Cell unit with cellulose disc of 65 mm diameter with pressure of 50 psi) with a cut-off membrane of 30 kDa. The ultra-filtrate was loaded and fractionated through Q-Sepharose (1.3 × 5 cm), which was pre-equilibrated with 20 mM Tris-HCl buffer, pH 8, at a flow rate of 3 mL/10 min. After washing with two column volumes of Tris buffer, the Q-Sepharose column-bound proteins were stepwise eluted with increasing NaCl concentrations in the same buffer. The proteins that were eluted with 0.1 M and 0.25 M NaCl were collected and extensively dialyzed against distilled water to remove NaCl, and then lyophilized to concentrate. The lyophilized pooled garlic lectin material was dissolved in sterile PBS, and Protein concentration were then measured by Bradford assay, in a microtiter format, following the published protocol. Later, the protein concentrations were adjusted to 1 mg/mL. The purity of the protein was assessed by reducing 12% SDS-PAGE following the standard procedure. The hemagglutination assay was performed with these samples to confirm the lectin activity. Commercially obtained ovalbumin (OVA) was prepared in sterile PBS at 1 mg/mL concentration.

### 2.4. Grouping of Animals for Immunization

The animals were grouped to study the adjuvant response in BALB/c mice, which were divided accordingly into four groups (*n* = 6) for intranasal and intradermal immunization. The selected mice were distributed randomly into groups based on their body weight. The average weights of the mice in each group were kept similar, mainly to ensure the equivalent response from each group of mice receiving the antigen. The first group was the vehicle control, where only saline was injected as a dose to stress the animals. The second group received ovalbumin (OVA); the third and fourth groups received ASA I and ASA II, respectively. All the antigens (OVA, ASA I, and ASA II) were administered in a dose-dependent manner by either the intranasal or intradermal route at the antigen concentration following the reported protocol [25].

### 2.5. Systemic (Intradermal) Immunization

**Figure 1:**
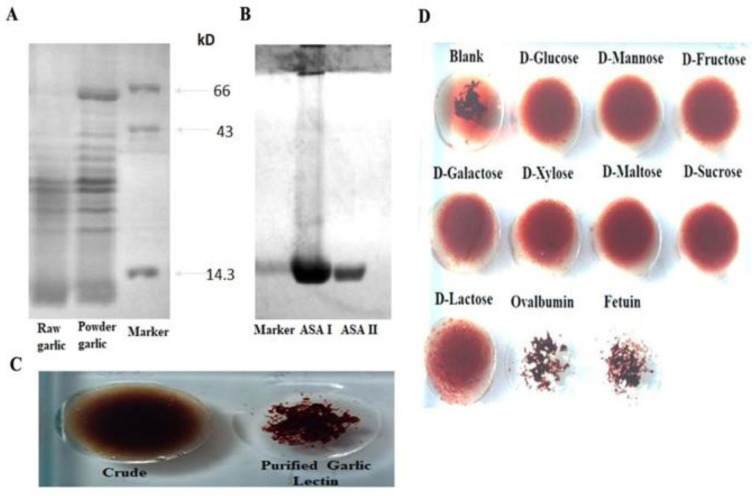
(**A**) SDS-PAGE protein profile of raw garlic and powdered garlic PBS extracts. (**B**) SDS-PAGE of raw garlic, purified lectins (ASA I and II), and molecular mass determination under reducing conditions with Coomassie brilliant blue staining. (**C**) Agglutinating activity with 1% trypsinized rabbit RBCs for the crude extract and purified garlic lectins. (**D**) Agglutination inhibition by mannose, glucose, and maltose, indicating the lectins’ sugar specificity.

The intradermal (i.d.) immunization schedule is represented in Figure 1 (top portion). The grouped mice (*n* = 6) obtained an intradermal injection of a constant 30 μL of 1 mg/mL of the respective antigen on days 0 and 7 to the dorsum of each ear; the control animal was treated (without the antigen) and administered only 30 μL of saline to stress the animal throughout the experiment. After day 14, all mice were sacrificed following the standard protocol, and blood was drawn by cardiac puncture. Blood was also collected from the treated and untreated groups by retro-orbital venipuncture at specific intervals (days 7 and 14).

### 2.6. Mucosal (Intranasal) Immunization Schedule

Intranasal (i.n.) administration of antigens was performed in groups of mice (*n* = 6), as represented in Figure 1 (bottom portion). Mice were immunized on days 1, 7, 14, 21, 28, 35, and 42 with one of the following: PBS, OVA, ASA I, and ASA II. All the samples were prepared in sterile PBS and were made up to the concentration of 1 mg/mL. Mice were moderately anesthetized through the exposure to a mild dose of diethyl ether and were carefully monitored during the intranasal administration of the antigen by heartbeat observation. Using a micropipette, 30 μg of OVA or garlic lectins (ASA I and II) were delivered in 30 μL of phosphate-buffered saline (PBS) (for dosage administration, 15 μL was applied slowly to each nostril and allowed for inhalation). After the antigen exposure regime, on day fifty, all mice were sacrificed following the standard protocol and blood was collected by cardiac puncture. Blood was drawn from the mice during specific intervals from the treated and untreated animals by retro-orbital venipuncture at days 14, 35, and 50.

### 2.7. Body Weights, Splenic, and Thymic Indices

All animals were maintained in an ambient environment during the experiment and were fed with a commercial mouse diet. The weight of individual mice was monitored at selected time intervals during the progress of the experiments before the administration of test doses by the i.d. or i.n. routes. The weight of the animals was noted at a selected period of intervals, and the body weights were recorded on the 14th day for the intradermal (i.d.) group, and on the 50th day for the intranasal (i.n.) group. The change in the animal weights reflects the effect of administrated antigens on animal physiology, which further enemarates growth or retardation.

The immunologically responding organs, like the spleen and thymus of the experimental animals, were isolated and collected separately after sacrificing the mice on the last day. The weights of the spleens and thymuses were recorded both in the treated and untreated groups. The splenic index and thymic index were calculated and were based on the spleen and thymus weights and the body weight. The thymic and splenic indices were calculated as follows: splenic index is measured as spleen weight (mg)/body weight (g), and thymic index is measured as thymus weight (mg)/body weight (g).

### 2.8. Adjuvant Activity of Garlic Lectin against OVA through Mucosal Immunization

The animals selected for studying mucosal adjuvant activity were immunized as per the schedule on days 1, 14, 21, 28, 35, and 42. The groups were administered with PBS (control), ovalbumin (OVA) alone (30 μg), or OVA (30 μg) blended with either one of Con A (30 μg), ASA I (30 μg), ASA II (30 μg), or RGE (30 μg). The mice were strained and were administered with intranasal dosing of 30 μL of each sample (15 μL was dosed slowly through each nostril) with the help of fine tips attached to a micropipette. The animals were exposed to a low dose of diethyl ether for partial anesthesia and were clutched in place until the liquid was inhaled completely. The serum was isolated from the blood samples collected from the treated and untreated groups on the 14th and 35th days prior to immunization. On day 50, all mice were sacrificed with an overdosing of anesthesia and the blood was collected with cardiac puncture.

### 2.9. Collection of Blood from Experimental Animals and Serum Separation

The animals were anesthetized, and blood samples were drawn from all the groups at specific intervals by retro-orbital venipuncture with the help of heparinized capillary tubes. On the last day, i.e., the 14th day for the intradermal group and the 50th day for intranasal group during the experiment schedule, the animals were sacrificed by an overdose of anesthesia followed by cardiac puncture. The heparinized bloods collected in the tubes were cotton plugged and kept at 24 °C for one hour for clotting. After, the tubes were centrifuged at 2500 rpm for 10 min in the refrigerator centrifuge with the temperature adjusted to 4 °C. The upper yellowish clear serum was pipetted to a new tube and stored at −20 °C; the same was used to measure the anti-lectin (ASA I and II)-specific IgG antibody response and the anti-ovalbumin (OVA)-specific IgG antibody response on the systemic, mucosal, and adjuvant experimental groups.

### 2.10. Detection of Anti-Lectin (ASA I and II) IgG and Anti-OVA-IgG Antibodies

The anti-lectin IgG and anti-OVA IgG responses on antigen administration through the systemic and mucosal routes was measured in the sera collected from the experimental group by ELISA. The detailed procedure for the experiment for the recognition of the IgG specific to garlic lectin (ASA I and II) and OVA in sera were described earlier [32]. Briefly, for the assay, 0.1 mg/mL of antigen samples were prepared in coating buffer (0.1 M carbonate-bicarbonate buffer, pH 9.6); the plates were then coated with 10 μg in 100 μL per well of samples and incubated at 4 °C overnight. After, the plates were washed with PBS containing 0.05% Tween (PBS-T) and the plates were further blocked with 2% gelatin prepared in 10 mM PBS and incubated at 37 °C for 2 h. Following incubation, the plates were washed and added with appropriately diluted serum samples as a source of primary antibody (mouse serum diluted 1:10 with a dilution buffer containing PBS-T with 1% BSA) and incubated at 4 °C overnight. The pooled serum derived from each individual animal of the groups was used as triplicate during the experiment. After the incubation, the plates were washed and incubated at 37 °C for 2 h by adding 100 μL/well of goat anti-mouse IgG conjugated to alkaline phosphatase (1:1000 dilution with PBS-T containing 1% BSA) as secondary antibody. Later, with extensive washing, the wells received 100 μL of AP substrate (p-nitrophenyl phosphate at 1 mg/mL prepared in 10% diethanol amine buffer, pH 9.4) and incubated for 20 min, and the reaction was arrested by the addition of 100 μL 3 N NaOH. The absorbance was read at 405 nm using microtiter plate reader.

### 2.11. Statistical Analysis

Data derived during the experiments are presented as the arithmetic mean and standard deviation. Significance and relative difference between the unpaired groups were identified by Student’s *t*-test; *p* < 0.05 was considered as statistically significant. For the measurement of significance within the same experimental group, a paired *t*-test was used to test for significance at different time points. All the statistical analyses were performed using SPSS software, version 10 (SPSS Inc., Chicago, IL, USA).

## 3. Results

### 3.1. Purification and Characterization of Garlic Lectins (ASA I and II)

The fractionated raw garlic extract on the Q-Sepharose chromatography separates ASA II and ASA I with step elution at 0.1 M and 0.25 M NaCl, respectively. The pooled eluted fraction was identified as ASA II and ASA I (in a ratio of 1:4 by weight), as judged by the hemagglutination activity and the glycocode assay. The SDS-PAGE profile of ASA I and ASA II, along with RGE, is shown in Figure 1; both appear as ~14.3 kDa proteins. The lymphoproliferative properties of ASA I and ASA II had demonstrated the lymphoproliferative properties in vitro and were in accordance with the previously reported observations of Clement et al. [15] on the characterization of garlic lectins.

### 3.2. Intradermal and Intranasal Immunization Regime

**Figure 2:**
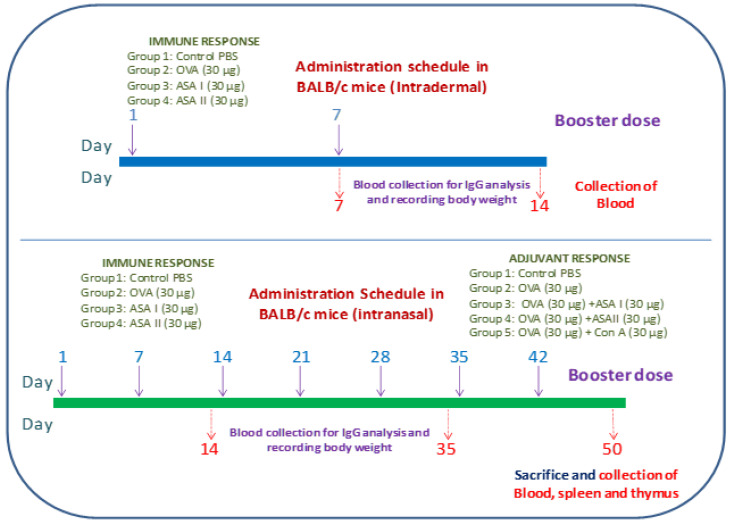
Systemic and mucosal administration schedule of garlic lectins in BALB/c mice model; Intradermal systemic immunization schedule. The animals were given the initial dose on day 1, and the subsequent booster dose was administered on day 7. Blood was drawn on days 7 and 14 for examining the immune response; Intranasal mucosal immunization schedule. The animals were given the initial dose on day 1, and the subsequent booster doses were administered on 7th, 14th, 21st, 28th, 35th, and 42nd days. Blood was drawn on days 7, 35, and 50 for measuring the immune response.

The experimental mice were administered with purified lectins in order to examine the humoral and adjuvant immune responses. The mice were grouped into four (*n* = 6) to receive antigens by both intradermal and intranasal immunization. The intranasal and intradermal immunization schedule for ASA I and ASA II for systemic, mucosal, and adjuvant immune responses was followed as illustrated in Figure 2.

### 3.3. Body Weights of Animals in the Intradermal and Intranasal Administration Groups

The weights of the individual experimental animals in both the treated and control groups were recorded. Mice body weights were measured prior to the administration of each antigen dose and the average animal weights in each were managed in the range of 25 ± 1 g. The body weights of the mice belonging to the experimental groups that were administered antigen by the intradermal route are presented in Table 1. There is no observed significant difference in the body weights of the mice in comparison with those of the control group. Though there is a marginal increase in the weight (~0.3 to 0.8 g) in both treated and control groups, the results are not found to be significant.

The body weights of the animals in each experimental group which were administered the antigen through the intranasal route are shown in Table 2. From day 0 to day 50 in the experiment, both the untreated control and the treated groups showed slight changes in the body weight, and these observed differences may due to the varied physiological and metabolic responses of the animals to the administered antigens in the respective experimental groups.

### 3.4. Anti-Garlic Lectin IgG Immune Response upon Systemic Administration

**Figure 3:**
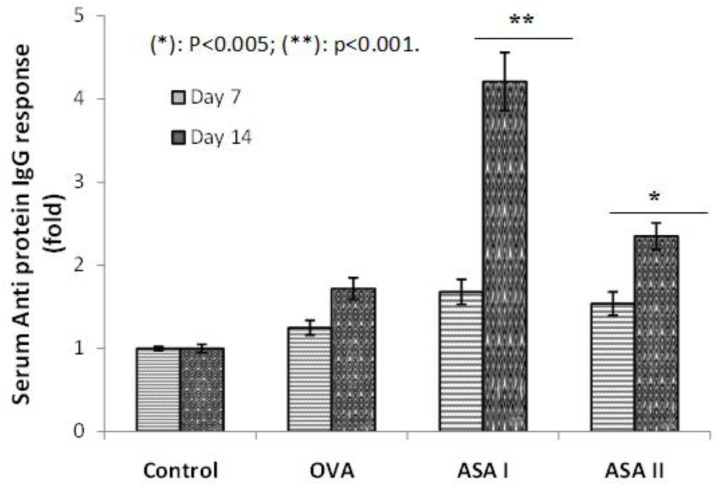
Serum IgG systemic immune response to ovalbumin (OVA) and garlic lectins (ASA I and II) after intradermal administration in BALB/c mice. Antigens (100 μL/well) were coated overnight, washed, blocked with BSA, and incubated with test serum. Detection with goat anti-mouse IgG-AP and PNPP substrate. The IgG response is represented as a fold increase in comparison with control, taking the absorbance of the control as 1. Panel indicates IgG immune response against OVA, ASA I, or ASA II as assessed by ELISA using the serum of experiment animals obtained at day 7 and day 14 by the intradermal route. Coated antigen amount: 10 μg/well; IgG serum dilution, 1:10 (volume: 100 μL); * *p* < 0.005; ** *p* < 0.001.

The systemic effect on the administration of OVA or garlic lectins (ASA I and II) in BALB/c mice through the intradermal route was studied for a targeted immune response without an adjuvant. The ovalbumin (OVA) is generally accepted as a weak antigen and served as control. The serum IgG response to the garlic lectins (ASA I and II) was compared to the anti-OVA IgG response and was considered as an indicator of the systemic immune response, as shown in Figure 3. The anti-OVA IgG immune response on day 7 and day 14 were almost cognate; however, both garlic lectins (ASA I and II) showed a considerable increase in anti-lectin IgG antibodies on day 14 in comparison to day 7. On day 14, the anti-lectin IgG antibodies titer was found to be ~4-fold increased, and found significant at *p* < 0.005 for ASA I. It was 2.5-fold higher for ASA II as compared to the IgG response to the OVA control.

### 3.5. Anti-Protein IgG Immune Response upon Mucosal Administration

**Figure 4:**
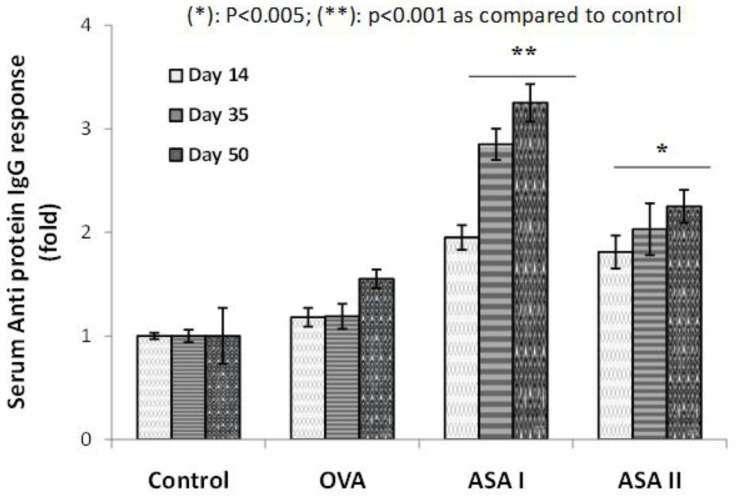
Mucosal immune (anti-OVA and anti-lectin) IgG response analyzed in the sera obtained from BALB/c mice after intranasal administration of antigens. Antigens (100 μL/well) were coated overnight, washed, blocked with BSA, and incubated with test serum. Detection of IgG antibody response by using goat anti-mouse IgG-alkaline phosphatase-conjugated antibody and *p*-nitrophenyl phosphate (PNPP) substrate. The IgG response is represented as a fold increase in absorbance over control, wherein the absorbance of the control was taken as 1. Coated antigen amount is 10 μg/well; primary antibody source, mice sera dilution was at1:10 (volume, 100 μL); * *p* < 0.005 vs. control; ** *p* < 0.001 vs. control. Each value is represented as mean ± S.D. (*n* = 3).

The weak antigen OVA and immunogenic garlic lectins were administered to BALB/c mice through the intranasal route to study the anti-protein IgG mucosal immune response. Serum containing protein-specific IgG antibodies were collected at different intervals of time from day 1 to day 50. The sera were used for measuring the anti-OVA and anti-lectin IgG immune responses, with the results represented in Figure 4. Ovalbumin (OVA) showed a very small increase in IgG antibodies specific to OVA from day 14 as compared to the control, which was not significant. This suggests that the anti-OVA response showed an increase after the second booster dose at day 50 (*p* < 0.01). The IgG response specific to garlic lectin (ASA I and II) is considerably higher and most significant as compared to the anti-OVA IgG response (*p* < 0.01). The anti-ASA I IgG response is evidently strong (2.5–3-fold increase), whereas in the case of anti-ASA II IgG, the response is ~2-fold higher as compared to the OVA group.

### 3.6. Splenic and Thymic Indices in Control and Treated Groups

**Figure 5:**
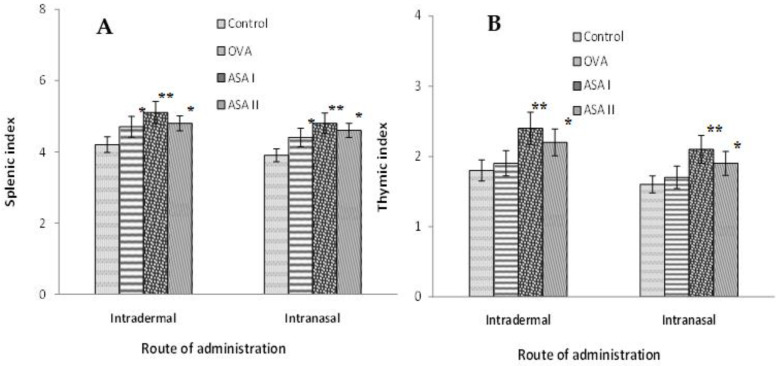
Splenic and thymic indices were measured in the control and test experimental groups after intranasal and intradermal immunization of OVA and garlic lectins in BALB/c mice. (**A**) Splenic index values for OVA, ASA I, or ASA II experimental groups on intradermal or intranasal administration of antigens. Spleen was collected on day 14 for intradermal groups and on day 50 for intranasal groups. Each value is represented as mean ± S.D. (*n* = 6); * *p* < 0.05; ** *p* < 0.01. (**B**) Thymic index values for OVA, ASA I, or ASA II experimental groups by intradermal or intranasal routes of antigen administration. Thymus was collected on day 14 for intradermal groups and on day 50 for intranasal groups.

After sacrificing the animals on the 14th day for the intradermal (i.d.) group, or the 50th day for the intranasal (i.n.) group, the spleens and thymuses were isolated. The weights of both spleens and thymuses were measured to obtain information on the possible stimulation of the thymus or spleen induced by the administered antigens (OVA and garlic lectins), the observed result of which is presented in Table 3. It is seen that the thymus and spleen weights were observed to be slightly increased in the garlic lectin-treated groups; however, a considerable or significant observable difference was not seen in the splenic or thymic weights between the OVA and the garlic lectin groups. The splenic and thymic indices are shown in Figure 5, panels A and B, respectively; the thymic and splenic indices are found to measure the differences accurately among the control and treated groups. The ASA I group significantly stimulates both the thymus and the spleen as compared to the control, OVA, and ASA II groups.

### 3.7. Humoral Adjuvant Property of Garlic Lectins (ASA I and II)

The mucosal adjuvant potential of garlic lectins (ASA I and ASA II) was assessed with a weak antigen OVA. The OVA was administered in combination with ASA I and II by the intranasal route and were examined for their ability to induce the humoral immune response to self and OVA. ASA I and ASA II were further analyzed for their effectiveness in interaction with immune cells to enhance the IgG antibody response against OVA in order to assess their adjuvanticity. The results of the body weights of the mice in the experimental groups of the control and those treated with OVA alone, OVA with ASA I, and OVA with ASA II were studied for their physiological effects on the growth of animals during the experimental regime. The anti-OVA IgG response for OVA alone and OVA with garlic lectins (ASA I and ASA II), Con A, and RGE were also assessed so to understand the humoral response and adjuvant potential of ASA I and ASA II, which were presented as follows:

#### 3.7.1. Body Weights of Experimental Animals during Mucosal Adjuvanticity Study

The experimental mice in the control and treated groups were monitored for body weights after the intranasal administration of the antigens. The change in body weights of the control group were compared with the OVA alone or the OVA with garlic lectins in the adjuvanticity study to mark the significant impact of the antigen administration in the growth and development of the animals. The results are presented in Table 4. There is no observed significant difference in the body weights of the mice in the test groups at day 0, 14, 35, and 50 as compared to the body weights of the mice in the untreated control group. Similarly, there is no observed significant difference in the body weights of the animals on day 50 vs. day 0 in each group. This justifies that the administered antigens did not demonstrated any growth retardancy in any of the experimental group.

#### 3.7.2. OVA-Specific IgG Response in the Garlic Lectin Adjuvanticity

**Figure 6:**
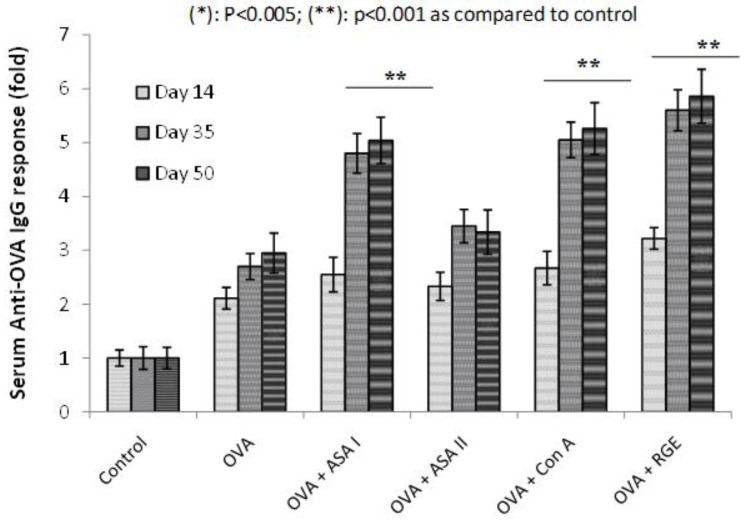
Ovalbumin (OVA)-specific IgG mucosal immune response to intranasal administration of OVA alone, or after OVA, co-administered with garlic components (ASA I or ASA II or RGE) or Con A by intranasal route at different times of administration. Coating antigen (10 μg OVA in 100 μL/well) overnight, washed, blocked with BSA, and incubated with test serum. Detection was with goat anti-mouse IgG-alkaline phosphatase conjugated antibody and PNPP substrate. The IgG response is represented as a fold increase taking the absorbance of the control as 1; serum dilution: 1:10; ** *p* < 0.001 vs. control. Each value is presented as mean ± S.D. (*n* = 3).

The adjuvanticity property of purified garlic lectins (ASA I and ASA II) was studied for the OVA antigen. OVA alone or OVA with ASA I/ASA II were administered by the intranasal route to the BALB/c mice in the experimental groups. Ovalbumin (OVA) was considered as an experimental weak antigen and the IgG response against OVA administered with garlic lectins was measured to determine the adjuvant property of ASA I and ASA II lectins. Comparing to the adjuvant effect of garlic lectins, Con A with Glc-/Man-specificity was used as a prototype lectin, and raw garlic extract (with all the components of raw garlic) was also considered so to understand and compare the adjuvanticity effect. The OVA-specific IgG response measured from the sera collected at different interval periods of the treatment (14th, 35th, and 50th day) is shown in Figure 6.

During the adjuvant experiment, in the experimental group where the intranasal administration of OVA was done along with ASA I and ASA II, the lectin ASA I group showed an increase in the anti-OVA IgG response on days 35 and 50 in comparison with the anti-OVA IgG response of the OVA group. Compared to the OVA-alone group, the OVA + ASA I group showed around a two-fold high (*p* < 0.005) OVA-specific IgG immune response (Figure 6). However, the OVA + ASA II group showed only a marginal increase (not significant) in comparison to the OVA-alone group. The OVA + Con A group showed almost identical observations in the IgG response as in OVA + ASA I. However, the OVA + RGE group showed the highest OVA-specific IgG response (marginally higher to ASA I and Con A groups).possibly, due to the synergetic stimulation of immune cells with garlic compounds.

### 3.8. Anti-OVA IgG Titer in Adjuvanticity Experiment in Control and Treated Groups

**Figure 7:**
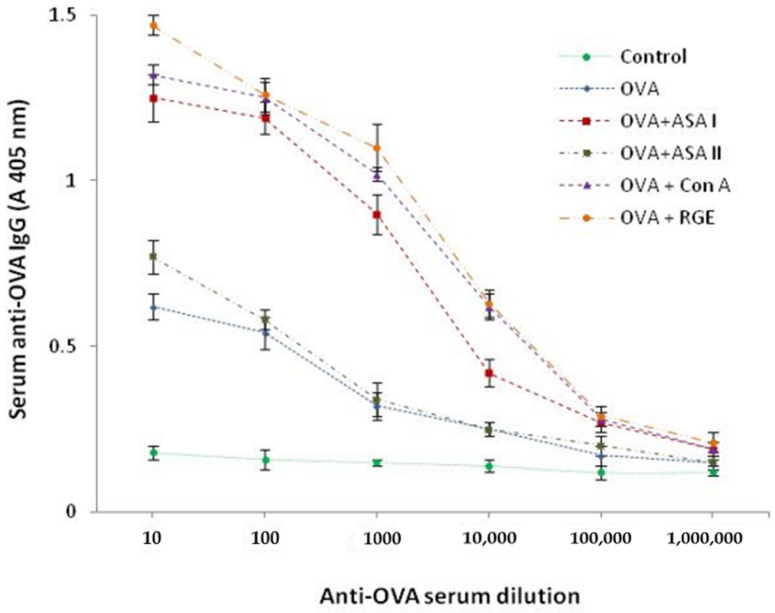
Anti-OVA IgG antibody titer (ELISA absorbance value at 405 nm) detected for sera obtained from untreated control and adjuvant treated experimental groups (OVA, OVA + ASA I, OVA + ASA II, OVA + Con A, OVA + RGE). Control and test proteins (100 μL/well; 0.1 mg/mL concentration) were coated overnight, washed, blocked with BSA, and were incubated with test serum. Detection with secondary antibody conjugate (goat anti-mouse IgG-AP) and PNPP substrate. The initial dilution of serum with 1:10 and was further serially diluted by 10-fold (10^−1^ to 10^−5^) using the dilution buffer.

The sera obtained from the adjuvanticity study groups was assessed for the anti-OVA antibody (IgG) response in the OVA-alone and adjuvant-treated groups (OVA + ASA I/ASA II/Con A/RGE) and was measured for its antibody titer, and the observations are presented in Figure 7. The IgG antibody titer in the sera obtained on the 50th day after the final sacrifice of the animals of both the control and treated groups was measured and compared for the assessment of adjuvanticity. It has been identified that the OVA-specific IgG antibody response was stronger in the OVA + ASA I as well as the OVA + Con A groups in comparison with the OVA and OVA + ASA II groups. The limit of antibody detection was observed at the dilution titer of 1:10,000 of serum in the case of the OVA + ASA I, OVA + Con A, and OVA + RGE groups. In the case of the OVA and OVA + ASA II groups, the antibody detection was found significant at a serum dilution of 1:100. These observations made during the antibody titer detection clearly emphasize that ASA I, Con A, and RGE significantly enhance the induction of IgG antibody production against the weak immunoantigen (OVA) and signifies that they stimulate the immune cells involved in humoral immunity as an adjuvant to increase the IgG immune response to the co-administered antigen OVA.

## 4. Discussion

The ingested dietary components should pass through the digestive tract, which is the largest immunological organ of the body, where dietary molecules interact with the immune cells. It possesses the largest interactive surface area through intestinal villi that are exposed to the external milieu, and thus confronted with the biggest immunoantigenic load, comprising pathogens, dietary molecules and proteins, and pathogens and commensal organisms [22]. The ‘gut’ immune system has the capability to distinguish foreign (toxic) proteins and nutrient proteins [9]. Gut-associated lymphoid tissue (GALT) is very much selective in recognizing dietary antigens; the unique gut immune microenvironment provides ambience to stimulate and regulate an immune response to food antigens by the suppression of immunocompetent cells.

Current information indicates that the administration of immunogenic antigen proteins by intradermal or intranasal routes induces vigorous anti-protein IgG immune responses [33,34]. The observations drawn in the present study demonstrated that garlic lectins (ASA I and ASA II) are effective immunogenic proteins which stimulate humoral antibody (IgG) responses following the systemic (intradermal) and mucosal (intranasal) administration of antigens. While the antigenic delivery of ASA I or ASA II induces a higher antibody titer on both the systemic and mucosal IgG immune response in comparison with OVA, the magnitude of the IgG response was found to be different for OVA, ASA I, and ASA II, and was very high for lectin ASA I, indicating its ability to interact efficiently to stimulate immune cells to induce response. The administered antigens had no toxic effect on the growth and development of the experimental animals, and no adverse effects were observed on the mice upon either intradermal or intranasal delivery. It may be recalled here with the report that the oral administration of garlic also resulted in the induction of significant immunogenicity in BALB/c mice [11]. It was observed that both the garlic lectin, ASA I and ASA II, groups showed a 3–4-fold increase in IgG antibody response on day 50 as compared to the control. Further, garlic lectins were found to be potent immunogenic proteins, and were found equivalent to phytohemagglutinin (lectin from red kidney bean) in immune recognition and stimulation [11].

In this study, it is observed that ASA I or ASA II showed reasonable immunogenic properties by inducing IgG immune response against self- and co-administered antigens, thus indicating that garlic lectins have intrinsic immunogenicity despite the absence of an adjuvant. Such antibodies produced were likely to be similar to ‘natural antibodies’ which have been reported for many dietary proteins and several plant lectins, including garlic lectin [35]. Human systems have a tendency to have natural antibodies circulating in the serum that have a characteristic broad reactivity and which appear to protect humans against a variety of pathogens not previously encountered. The presence of these food proteins or lectin-triggered natural antibodies in human circulation prior to viral or bacterial infections were of great importance in providing infection tolerance and resistance to pathogenic diseases [36].

OVA is well reported in studies as an antigen with poor immunogenic properties [16,33]. The results from the present study indicate the same with the observed low anti-OVA IgG antibody titer and a significantly high anti-ASA I IgG (~2.5- to 3-fold) immunogenic response in comparison with the reference antigen (OVA), suggesting that ASA I is a strong immunogen. Anti-ASA II IgG response (~1.3- to 2-fold) showed moderately higher IgG response than OVA, which suggests that ASA II is also a weak immunogenic protein. Since ASA II showed around a three-fold lower binding efficiency to glycan structures than ASA I [15,31], this confirms the interrelationship of glycan-binding ability to possible immunogenic properties. Many reports suggest that the food lectins ingested through the diet will efficiently interact with the intestinal villous enterocytes based on their glycan recognition. Most of these lectins will cross the intestinal barrier and enter into the systemic circulation, where they encounter immune cells to activate the humoral immune response by inducing lectin-specific IgG responses. The dietary lectins entering the circulation via uptake across the Peyer’s patch for internalization through follicle-associated epithelium instigates the intestinal IgG and IgA immune responses in the intestinal-associated lymphoid follicles [21,37,38]. The interactive receptors in gut cells, to which lectins bind and interact for internalization, are not well known, but may appear to be the key factors in determining the lectin-induced mucosal immunogenicity [39].

The presented results in the study explore the adjuvanticity potential of lectins from garlic bulbs (ASA I and ASA II) which are co-administered with weak immunogenic antigen OVA through the intranasal route of administration. Garlic lectins exhibit an effective adjuvant property by showing a considerable increase in the OVA-specific IgG antibody titer response in the sera obtained from the OVA + ASA I group as compared to OVA-alone and OVA + ASA II groups. The observed results clearly indicate that among the garlic lectins, ASA I possesses significant adjuvant properties. RGE administered with OVA showed a slightly higher IgG antibody response than purified ASA I; this might be due to the possible synergistic adjuvant effects of the active garlic components (organosulfur compounds and fructans) in the RGE or the aged garlic extract [32,40,41]. Many proteins with an affinity for eukaryotic cell surface molecules are weak mucosal immunogens in mice. Different routes of administration may result in varied immune responses [38,42], as it is likely there may be possible differences in the immune cells present in the nasal-associated lymphoid and gut-associated lymphoid tissues [39].

**Figure 8:**
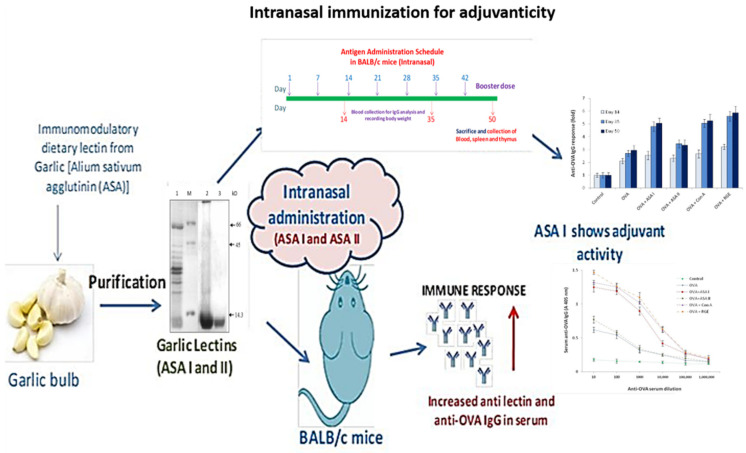
Illustration of the immune and adjuvant response of garlic lectins (ASA I and ASA II) on intradermal and intranasal administration in BALB/c mice. This explains the ability of garlic lectin, ASA I, as potential mucosal adjuvant for delivering and eliciting immune response against a weak antigen such as ovalbumin, through intranasal administration.

Garlic bulb lectins, specifically ASA I, exhibit a strong mucosal and systemic immune response by inducing anti-lectin IgG antibodies by both intradermal and intranasal routes of administration. ASA I also has the ability to produce a marked stimulation and proliferation of splenocytes and thymocytes [43,44]. In order to enhance the efficacy of weakly immunogenic ‘subunit’ vaccines (recombinant), there is an unmet need for specific adjuvants and targeted delivery systems that are efficacious when given mucosally (either by oral or nasal routes) [45,46]. ASA I, has all the properties to function as a mucosal adjuvant and can boost the humoral immune response to co-administered weak immunogenic antigens (Figure 8). This can be a potential dietary adjuvant for future consideration in targeted delivery by weak antigens for immunization through mucosal administration.

The observed results in our study demonstrate that garlic lectins (at 30 µg dose by i.n. administration) elicited humoral immunogenic response by inducing the increase in serum IgG titer in BALB/c mice, thereby revealing their intrinsic immunogenicity. Further, garlic lectins displayed humoral adjuvant immune response for the poor immunogenic antigen OVA (a reference model weak antigen), as is evident by the observed increase in the serum anti-OVA IgG antibody on days 35 and 50. The thymic and splenic indices of the garlic lectins administered through the intranasal route in the mice group were significantly higher than that of control or OVA groups. All in all, it appears that the intranasal administration of garlic lectins leads to the production of natural antibodies (serum IgG), which acts as mucosal adjuvants for the delivery of weak antigens. Therefore, garlic lectins (especially ASA I) are dietary immune system boosters and have the potential to stimulate immune cells involved in humoral immunity and are of great use as mucosal adjuvants for various experimental antigens (oral or nasal vaccine candidates with weak mucosal immunogenicity) in future studies on humans. The studies have some limitations in deciphering the mechanisms of mucosal immunogenicity through the activation immune cells and in understanding the impact of specific cytokine signaling. Further, it will require additional studies with a dose-dependent adjuvant and lectin combination so to optimize the garlic lectins for mucosal antigen delivery. However, the present study is the first proof identifying garlic lectins as a mucosal adjuvant to deliver antigens efficiently through oral or nasal mucosal routes. In addition, it has ability to activate immune cells to boost immune response and has no side effects with the present chemicals or with other immunoadjuvants. Hence, garlic lectins are promising future potential biological adjuvants of choice for antigen delivery through mucosal routes for efficient immunogenicity.

## Figures and Tables

**Table 1:** Body weights of mice at different intervals of experiment during intradermal (i.d.) administration for systemic response.

Group ^a^	Body Weights ^b^ (g), mean ± SD		
	Day 1	Day 7	Day 14 ^c^
Control	23.47 ± 1.62	23.70 ± 1.75	24.10 ± 2.32
OVA	24.26 ± 2.47 *	24.48 ± 1.91	24.74 ± 2.98 *
ASA I	24.62 ± 2.12	24.57 ± 1.72	24.68 ± 2.24
ASA II	24.53 ± 1.63	25.03 ± 1.29	25.36 ± 2.13 *

^a^ *n* = 6 animals in each group; OVA, ovalbumin; ASA, *Allium sativum* agglutinin. ^b^ the body weight is presented mean ± SD of mice in a group. ^c^ Versus day 1; * Significant at *p* < 0.005.

**Table 2:** Body weights of mice at different intervals of experiment during intranasal administration for mucosal response.

Group ^a^	Body Weights ^b^ (g), mean ± SD			
	Day 1	Day 14	Day 35	Day 50 ^c^
Control	25.33 ± 3.29 *	25.73 ± 4.50	26.17 ± 3.57	27.03 ± 2.74 *
OVA	25.58 ± 1.55	26.24 ± 2.41	26.94 ± 2.13	26.80 ± 2.56
ASA I	25.68 ± 1.39 *	25.24 ± 1.86	25.28 ± 1.36	25.22 ± 1.59 *
ASA II	25.44 ± 1.26	25.66 ± 0.85	25.56 ± 0.89	25.38 ± 1.33

^a^ *n* = 6 animals in each group; OVA, ovalbumin; ASA, *Allium sativum* agglutinin. ^b^ The body weight is expressed as mean weight of animals in a group. ^c^ Versus day 1; * Significant at *p* < 0.005.

**Table 3:** Weights of spleen and thymus of BALB/c mice by intradermal (i.d.) and intranasal (i.n.) administration of antigen (OVA, ASA I and II) samples.

Group ^a^	Intradermal Route ^b^		Intranasal Route ^c^	
	Thymus Weight (mg) mean ± SD	Spleen Weight (mg) mean ± SD	Thymus Weight (mg) mean ± SD	Spleen Weight (mg) mean ± SD
Control	43.45 ± 2.13	106.83 ± 07.99	40.42 ± 4.63	107.47 ± 9.91
OVA ^d^	46.83 ± 6.21	131. 95 ± 08.97	42.61 ± 3.52	116.43 ± 7.64
ASA I ^d^	55.32 ± 2.84	138. 56 ± 06.13	49.98 ± 5.84	128.89 ± 15.39
ASA II ^d^	51.27 ± 3.80	128.45 ± 10.12	44.12 ± 2.71	117.18 ± 12.05

^a^ *n* = 6 in each treated group; OVA, ovalbumin; ASA, *Allium sativum* agglutinin. ^b^ Spleen and thymus were collected for weight on day 14. ^c^ Spleen and thymus were collected for weight on day 50. ^d^ Versus the control (*p* < 0.01).

**Table 4:** Body weights of BALB/c mice at different intervals of time after antigen administration by intranasal route in adjuvanticity study.

Group ^a^	Body Weights (g), mean ± SD			
	Day 0	Day 14	Day 35	Day 50 ^b^
Control	23.26 ± 2.06	25.35 ± 2.15	24.29 ± 1.39	24.76 ± 1.86
OVA	23.93 ± 1.47	23.84 ± 2.17	24.47 ± 2.04	24.38 ± 2.89
OVA + ASA I	24.68 ± 2.08	24.12 ± 1.73	24.45 ± 2.23	23.89 ± 2.68
OVA + ASA II	23.47 ± 1.91	24.57 ± 2.09	25.17 ± 1.82	25.89 ± 2.13
OVA + Con A	24.68 ± 1.29	23.79 ± 1.87	24.46 ± 2.81	24.29 ± 1.78
OVA + RGE	24.67 ± 1.42	24.72 ± 1.35	25.97 ± 1.85	25.96 ± 2.46

^a^ *n* = 6 in each group; intranasal administration of each antigen at 30 μg in 30 μL volume; OVA, ovalbumin; Con A, concanavalin A; RGE, raw garlic extract. ^b^ Versus day 0; not significant at *p* < 0.05.

## Data Availability

All data are available within this publication.

## Sample Availability

Samples of the compounds are available from the authors.

## Abbreviations

AP: alkaline phosphatase; ASA: *Allium sativum* agglutinin; BALB/c: albino laboratory breed c-type mice; Con A: concanavalin A; ELISA: enzyme-linked immunosorbent assay; GALT: gut-associated lymphoid tissue; IgE: immunoglobulin E; IgG: immunoglobulin G; i.d.: intradermal; i.n.: intranasal; OVA: ovalbumin; PHA: phytohemagglutinin; PBS: phosphate-buffered saline; PNPP: *p*-nitrophenyl phosphate; RGE: raw garlic extract.
